# Optimization of alkaline pretreatment and enzymatic hydrolysis for the extraction of xylooligosaccharide from rice husk

**DOI:** 10.1186/s13568-018-0645-9

**Published:** 2018-07-16

**Authors:** Nuntawat Khat-udomkiri, Bhagavathi Sundaram Sivamaruthi, Sasithorn Sirilun, Narissara Lailerd, Sartjin Peerajan, Chaiyavat Chaiyasut

**Affiliations:** 1 Innovation Center for Holistic Health, Nutraceuticals, and Cosmeceuticals, Faculty of Pharmacy, Chiang Mai University, Chiang Mai, 50200 Thailand; 2 Department of Physiology, Faculty of Medicine, Chiang Mai University, Chiang Mai, 50200 Thailand; 3 Health Innovation Institute, Chiang Mai, 50200 Thailand

**Keywords:** Xylooligosaccharide, Rice husk, Xylan, Alkaline pretreatment, Enzyme hydrolysis, FTIR

## Abstract

Rice husk (RH) is the major agricultural waste obtained during rice hulling process, which can be a sustainable source of xylooligosaccharide (XOS). The current study deals with the production of XOS from Thai rice husk using alkaline pretreatment and enzyme hydrolysis method. The response surface methodology consisted of central composite design and Box–Behnken design was employed to achieve the maximum response in alkaline pretreatment and XOS production, respectively. The optimum conditions for alkaline pretreatment to recover maximum xylan yield were 12–18% of alkaline concentration, the temperature at 110–120 °C, and steaming time for 37.5–40 min. The FTIR results suggested that the extracted sample was the xylan fraction. The maximum XOS production of 17.35 ± 0.31 mg XOS per mL xylan was observed in the run conditions of 6.25 mg enzyme per g xylan, 9 h of incubation time, and 5% of xylan. The results revealed that the xylan extracted from RH by using an effective base couple with the steam application and the enzymatic hydrolysis help to maximize the yield of XOS, which can be further used in functional foods and dietary supplements.

## Introduction

Thailand is one of the leading rice producer and exporter. As per the US Department of Agriculture’s Foreign Agricultural Service report, Thailand may produce about 20.4 million tons of rice during 2017–2018. Rice husk (RH) is the major agricultural waste obtained during rice hulling process, and the clearance of agricultural waste by firing cause air pollution. The burning of rice straw in Thailand was attributed to the release of 0.18% of greenhouse gas (Gadde et al. 2009).

RH is composed of cellulose (25%), hemicellulose (25%), lignin (20%), ash (17%), and crude protein (3%) (Ugheoke and Mamat 2012). The major reducing sugar after acid hydrolysis of rice husk is composed of 17.35% of xylose and 4.42% of glucose (Banerjee et al. 2009). Xylooligosaccharide (XOS) is the molecule containing straight chains of xylose connected by β-1,4-glycosidic linkage. In general, linked minimum of 2–20 xylose molecules are consider as XOS (Vázquez et al. 2000). XOS is well known prebiotic molecule that can selectively support the growth and stimulate the activity of beneficial gut microbiota, particularly *Bifidobacterium* spp. thereby it confers the health benefits to host (Finegold et al. 2014; Gibson and Roberfroid 1995). XOS reported for anti-cancer, anti-microbial, antioxidant, anti-allergic, anti-infection, anti-inflammatory activities, immunomodulatory, and cholesterol-lowering property (Aachary and Prapulla 2011; Mumtaz et al. 2008). Thus, XOS has been accepted as nutraceutical and feed additive.

The conversion of agricultural waste into useful product reduce the problem of management, treatment, and disposal of agricultural residues. The lignocellulosic rich agricultural residues such as corn cob (Samanta et al. 2012a; Boonchuay et al. 2014), corn stalks (Ergues et al. 2012), sugarcane bagasse (Jayapal et al. 2013), cotton stalks, wheat straw, sunflower stalks, tobacco stalks (Akpinar et al. 2009a), pigeon pea stalks (Samanta et al. 2013), and green coconut husks (Jayapal et al. 2014) have been used as raw material for XOS production.

Autohydrolysis, chemical process (acid or alkaline solution treatment), and chemical pretreatment for xylan extraction couple with enzymatic hydrolysis is the possible way to extract XOS from lignocellulosic substrates (Qing et al. 2013). Autohydrolysis is the process for XOS production by heating the lignocellulosic materials with water in specific equipment under controlled condition, while acid hydrolysis is cost effective. Both the techniques produce XOS contaminated with other undesirable substances like lignin, monosaccharides, and furfural (an aldehyde of furan), which cause serious adverse effects like respiratory irritation, lung congestion, hyperplasia, kidney and olfactory epithelial damage, oedema, and inflammation. So further purification process is required to remove the unwanted substances in the product. Nowadays, chemical pretreatment for xylan extraction followed by enzymatic hydrolysis to obtain XOS is an often-preferable method, due to the cost-effective and reduced production time. The alkaline extraction is the important practice to separate xylan from other lignocellulosic materials, which further enhances the efficiency of the enzyme during XOS production (Carvalho et al. 2013; Samanta et al. 2015). In addition, alkaline pretreatment can improve substrate digestibility, the major desired characteristic in XOS production by enzymatic hydrolysis (McIntosh and Vancov 2011).

The current study was aimed to optimize the conditions to achieve high xylan yield in alkaline pretreatment from RH and XOS production by enzymatic hydrolysis.

## Materials and methods

### Materials

The rice husk (RH) was obtained from local organic farm in Chiang Mai province, Thailand. The food grade 1,4 beta xylanase (Pentopan™ MonoBG) was purchased from Novozymes, Denmark. Xylooligosaccharide standards including xylobiose, xylotriose, xylotetraose, and xylopentaose were obtained from Megazyme, Bray, Ireland. Xylose and arabinose standards were obtained from Wako Pure Chemical Industries, Osaka, Japan. The ion exclusion chromatography column, Shodex SUGAR SH1011, was purchased from Showa Denko K.K., Tokyo, Japan. Other chemicals used in the study were of analytical grade.

### Rice husk preparation

The RH was dried at 50 °C for 12 h, and powdered by the mechanical blender. The milled RH was sieved through 0.595 mm size (No. 30) siever, and stored at room temperature until use.

### Optimization of alkaline pretreatment of RH, and XOS production by RSM

Response surface methodology (RSM) and central composite design (CCD), and RSM and Box–Behnken design (BBD) were used to optimize the condition for alkaline pretreatment of RH, and XOS production using the statistical software package Design Expert, version 10.0., respectively (Stat-Ease Inc., Minneapolis, MN, USA). The basics and statistical analyses of RSM, and its applications in designing the experiment has reported previously (Woraharn et al. 2015, 2016; Chaiyasut et al. 2017). The recovery of xylan was the desired response after RH pretreatment. The concentration of alkaline (6–18%), steaming time (15–45 min), and steaming temperature (80–120 °C) have been selected as variable factors to achieve high yield of xylan as per the previous literature (Jayapal et al. 2013; Samanta et al. 2012a, b, 2013). A sum of 20 independent experiments composed of 14 combinations and six center point replicates were performed for alkaline pretreatment (Table 1), and 17 independent experiments performed XOS optimization. The following equation was used in CCD and BBD model.

1 $${\text{Y}} =\upbeta_{0} +\upbeta_{1} {\text{X}}_{1} +\upbeta_{2} {\text{X}}_{2} +\upbeta_{3} {\text{X}}_{3} +\upbeta_{11} {\text{X}}_{1}^{2} +\upbeta_{22} {\text{X}}_{2}^{2} +\upbeta_{33} {\text{X}}_{3}^{2} +\upbeta_{12} {\text{X}}_{1} {\text{X}}_{2} +\upbeta_{13} {\text{X}}_{1} {\text{X}}_{3} +\upbeta_{23} {\text{X}}_{2} {\text{X}}_{3}$$

where Y is the predicted response, β_0_ is model constant, βi (β_1–3_) is linear coefficients, βii (β_11–33_) is quadratic coefficients, βij (i.e. β_12_) is cross product coefficients; X_1_, X_2_ and X_3_ are independent variables.

**Table 1:** The variable factors and predicted and observed xylan recovery

Std	Run	Factors			Xylan recovery	
		X_1_: alkaline concentration (%w/v)	X_2_: steaming time (min)	X_3_: steaming temperature (°C)	Observed value (%)	Predicted value (%)
14	1	0 (12)	0 (30)	1.682 (133.64)	54.49 ± 0.61	52.66
13	2	0 (12)	0 (30)	− 1.682 (66.36)	11.71 ± 0.39	12.36
9	3	− 1.682 (1.91)	0 (30)	0 (100)	5.19 ± 1.27	7.73
7	4	− 1 (6)	1 (45)	1 (120)	44.39 ± 3.42	44.60
15	5	0 (12)	0 (30)	0 (100)	16.24 ± 0.53	13.87
17	6	0 (12)	0 (30)	0 (100)	15.07 ± 2.24	13.87
6	7	1 (18)	− 1 (15)	1 (120)	8.37 ± 0.42	13.94
16	8	0 (12)	0 (30)	0 (100)	13.67 ± 1.31	13.87
18	9	0 (12)	0 (30)	0 (100)	17.20 ± 4.30	13.87
1	10	− 1 (6)	− 1 (15)	− 1 (80)	8.39 ± 1.76	10.29
20	11	0 (12)	0 (30)	0 (100)	10.69 ± 0.01	13.87
10	12	1.682 (22.09)	0 (30)	0 (100)	14.89 ± 1.38	11.17
3	13	− 1 (6)	1 (45)	− 1 (80)	3.87 ± 0.54	− 0.87
2	14	1 (18)	− 1 (15)	− 1 (80)	10.86 ± 4.08	11.49
11	15	0 (12)	− 1.682 (4.77)	0 (100)	11.81 ± 0.62	7.97
12	16	0 (12)	1.682 (55.23)	0 (100)	24.14 ± 3.98	26.79
5	17	− 1 (6)	− 1 (15)	1 (120)	23.42 ± 2.09	22.45
4	18	1 (18)	1 (45)	− 1 (80)	9.92 ± 0.66	11.72
19	19	0 (12)	0 (30)	0 (100)	10.16 ± 1.19	13.87
8	20	1 (18)	1 (45)	1 (120)	48.55 ± 2.12	47.49

For the optimization of XOS production, the enzyme concentration (X_1_) (mg/g xylan), incubation time (X_2_) (h), and xylan concentration (X_3_) (%w/v) were acted as a variable factor while the concentration of XOS as a desirable outcome. The range of enzyme concentration, incubation time, and xylan concentration are 6–12 h, 15–45 min, and 80–120 °C, respectively (Chapla et al. 2012). The experiments were carried out in triplicates.

### Alkaline pretreatment of rice husk

The alkaline pretreatment to RH was carried out in duplicate according to Samanta et al. (2012a). Briefly, RH powder and alkaline solution (1.91–22.09% NaOH depended on the CCD model) was mixed at the ratio of 1:10 (at different alkaline concentration), and subjected to steaming process at various temperature for various duration based on the CCD model. After incubation, the solution was centrifuged at 5000 rpm for 20 min, and the supernatant was acidified with glacial acetic acid until the solution reached pH 5. Then, three volumes of 95% ice-cold ethanol were added to precipitate the xylan fraction and centrifuged at 4480×*g* for 10 min at 4 °C. The xylan precipitate was collected and dried at 55–65 °C until reach the constant weight. The dried pellet was weighed and stored at room temperature. The exact and relative yield of xylan was calculated according to formula 2.

2 $${\text{Xylan recovery (\%)}} = {\text{Dry weight of extracted xylan (g)}}/{\text{Weight of the sample (g)}} \times 100$$

The optimum condition to recover maximum xylan yield was used for the bulk xylan production and further analysis.

### Analysis of extracted xylan

Hemicellulose quantification of extracted xylan was carried out according to the method of National Renewable Energy Laboratory (NREL) (Sluiter et al. 2008). The hydrolysate was filtered through 0.2 μm syringe filter to remove the residues and then determined by HPLC. The sugar sample was subjected to HPLC equipped with refractive index detector (Model 2414, Waters Corporation) using Shodex SUGAR SH1011 column. The analytical column was maintained at 60 °C. The samples were eluted with 0.05 M sulfuric acid with the flow rate of 1.0 mL/min. The concentration of sugars was determined using peak area of the standard (a mixture of xylose, mannose, and galactose), and the concentration of each sample was expressed as XGM (xylan + galactan + mannan) content (%) dry hemicellulose basis (Gao et al. 2014).

The functional group identification of alkaline extracted xylan was done using Fourier transform infrared spectrophotometer (Thermo Nicolet, Nexus 470 FT–IR) at spectral range of 400–4000, 4 cm^−1^ resolutions, and DTGS with a KBr window detector. One mg of alkaline extracted xylan was used for FTIR analysis (Samanta et al. 2012a).

### Enzyme assay

The β-1,4-xylanase activity of commercial enzyme was carried out by using 1% birchwood xylan (Sigma, St. Louis, MO, USA) solution in citric acid—Na_2_HPO_4_ buffer (pH 6.0) as substrates. An equal volume of both commercial xylanase solution and substrate was incubated at 50 °C for 10 min (Bailey et al. 1992). At the indicated interval, the reaction was stopped by adding 3,5-dinitrosalicylic acid (DNS) solution and reducing sugar was quantified (Miller 1959). One unit of the xylanase activity (U) was liberated as the amount of xylanase that released 1 μmol of reducing sugar from the substrate xylan per min at pH 6.0, 50 °C.

### Enzymatic hydrolysis of alkaline extracted xylan and XOS determination

Enzymatic hydrolysis of rice husk xylan was done by adding 1 mL of commercial xylanase solution in 9 mL of alkaline extracted xylan solution in 50 mM citric acid-Na_2_HPO_4_ buffer (pH 6.0), and incubated at 50 °C for various time interval based on BBD model. After incubation reaction was arrested by placing the reaction tube in boiling water bath for 5 min. Then, the XOS mixtures were centrifuged at 6000 rpm for 10 min and filtered through 0.2 μm syringe filter to remove the residues. The samples were quantified by HPLC equipped with refractive index detector (Model 2414, Waters Corporation) using Shodex SUGAR SH1011 column. The samples were eluted with 0.05 M sulfuric acid with flow rate of 0.8 mL/min. The concentration of sugars was determined using peak area ratio of the mixture xylooligosaccharide standards including xylobiose (X_2_), xylotriose (X_3_), xylotetraose (X_4_), and xylopentaose (X_5_). Glycerol was used as the internal standard. The xylooligosaccharide concentration of samples was expressed as mg XOS/mL xylan.

### Statistical analysis

The alkaline pretreatment of rice husk and enzymatic hydrolysis of alkaline pretreated xylan were carried out in duplicates and triplicates, respectively. All values were expressed as mean ± SD. The difference between the group means was analyzed by one-way analysis of variance (ANOVA). The differences were considered significant at *P *< 0.05.

## Results

### Optimization of alkaline pretreatment of RH for xylan recovery

The effect of alkaline concentration (X_1_), steaming time (X_2_), and steaming temperature (X_3_) of RH on the yield were determined by three factors central composite design. Twenty independent experiments with six center points were performed. The predicted recovery of all the center points (X_1_ = 12%; X_2_ = 30 min; X_3_ = 100 °C) were 13.87%, whereas the actual experimental values were varied. About 16.24 ± 0.53, 15.07 ± 2.24, 13.67 ± 1.31, 17.20 ± 4.30, 10.69 ± 0.01, and 10.16 ± 1.19% of recovery was observed in the run numbers 5, 6, 8, 9, 11, and 19, respectively (Table 1).

The analysis of variance, regression coefficients, and response surface plots were carried out using design expert. The analysis of variance of a quadratic model for the yield of alkaline extracted xylan was shown in Table 2.

**Table 2:** Analysis of variance for quadratic model of alkaline treatment of rice husk

Source	Sum of squares	Mean square	F-value	*P*-value
Model	3774.96	419.44	27.80	< 0.0001
Residual	150.88	15.09		
Lack of fit	108.83	21.77	2.59	0.1600
Pure error	42.05	8.41		
Cor total	3925.84			

Recovery yield: R^2^ = 96.16%, R^2^ (adjacent) = 92.70%

The regression model for xylan extraction was significant (*P *< 0.0001) with appreciable R^2^ (96.16%) and adjusted R^2^ (92.70%), and non-significant lack of fit (*P *= 0.1600). The results suggested that the model equation was adequate for the prediction of recovery yield of alkaline extracted xylan from RH. The CCD-generated a quadratic equation for xylan recovery yield (Y) was as follows:

3 $${\text{Y}} = 1 7 4. 5 9 2+ 2. 2 8 5 {\text{X}}_{ 1} - 3. 1 1 3 {\text{X}}_{ 2} - 3. 2 8 6 {\text{X}}_{ 3} - 0.0 4 3 {\text{X}}_{ 1}^{ 2} + 0.00 6 {\text{X}}_{ 2}^{ 2} + 0.0 1 6 {\text{X}}_{ 3}^{ 2} + 0.0 3 2 {\text{X}}_{ 1} {\text{X}}_{ 2} - 0.0 20{\text{X}}_{ 1} {\text{X}}_{ 3} + 0.0 2 8 {\text{X}}_{ 2} {\text{X}}_{ 3} \ldots$$

The estimated regression coefficients revealed that the steaming temperature (*P *< 0.0001) and steaming time (*P *= 0.0003) were had a significant impact in alkaline pretreatment and xylan yield (Table 3).

**Table 3:** Estimated regression coefficients for alkaline pretreatment from rice husk

Term	Estimated parameters	*P*-value
Alkaline concentration	2.285	0.3541
Steaming time	− 3.113	0.0003
Steaming temperature	− 3.286	< 0.0001
Alkaline concentration × steaming time	0.032	0.0648
Alkaline concentration × steaming temperature	− 0.020	0.1079
Steaming time × steaming temperature	0.028	0.0001
Alkaline concentration × alkaline concentration	− 0.043	0.1573
Steaming time × steaming time	0.006	0.2531
Steaming temperature × steaming temperature	0.016	< 0.0001

**Fig. 1:**
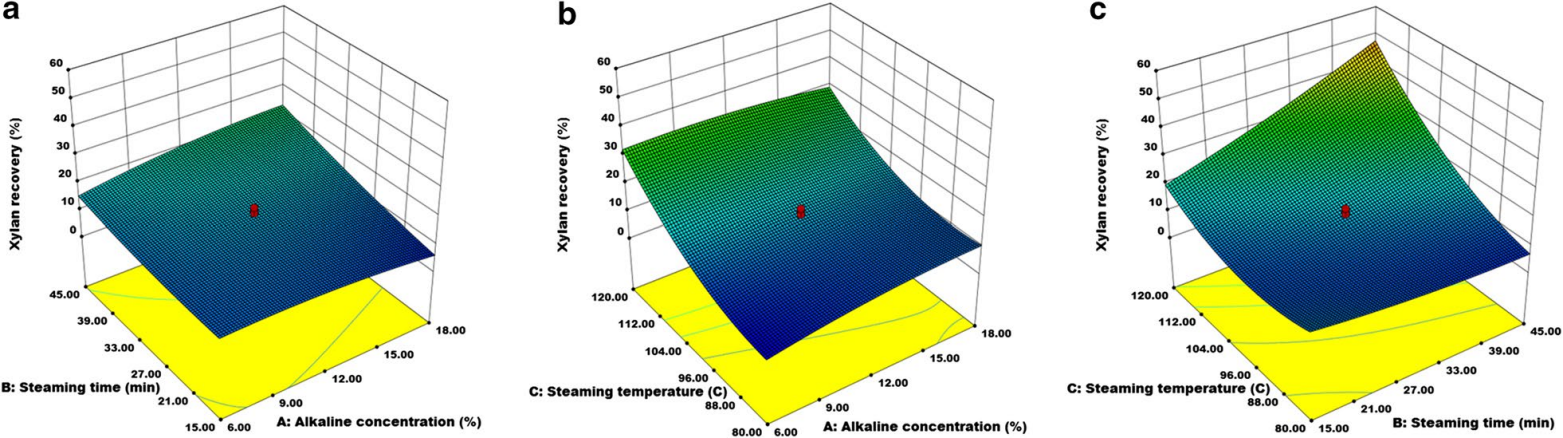
Response surface plot for alkaline pretreatment of rice husk describing the interaction of independent variables such as alkaline concentration, steaming temperature and steaming time. **a** Effect of alkaline concentration and streaming time, **b** effect of alkaline concentration and streaming temperature, and **c** effect of streaming time and streaming temperature

In addition, the steaming temperature and steaming time also exhibited the synergistic effect (*P *= 0.001) during alkaline pretreatment (Fig. 1). The results suggested that both the factors, steaming time and temperature, have elevated the effect on the recovery of alkaline extracted xylan. The maximum xylan yield was, as per Table 1, observed in the run conditions 1, 4, and 20 with the yield of 54.49 ± 0.61, 44.39 ± 3.42, and 48.55 ± 2.12%, respectively. The optimum conditions for alkaline pretreatment to recover maximum xylan yield were 12–18% of alkaline concentration, the temperature at 110–120 °C, and steaming time for 37.5–40 min.

### FTIR analysis

**Fig. 2:**
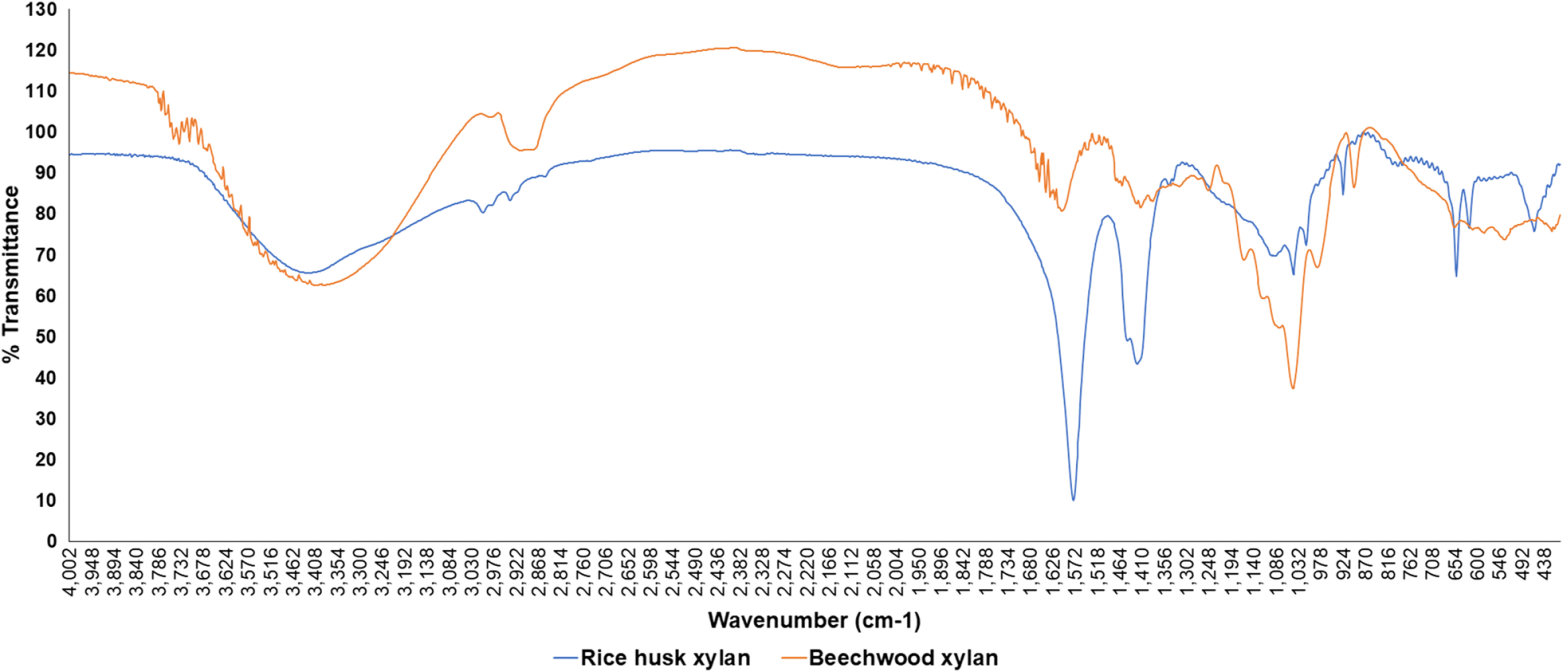
The FTIR pattern of extracted xylan

The extracted xylan was analyzed by FTIR. The FTIR fingerprint pattern was compared with standard beechwood xylan (Fig. 2). FTIR spectra of the sample were closely similar to the standard xylan fraction with high bands at 1600–1000 cm^−1^. The broad bands were noticed between 3600 and 3200 cm^−1^. The results clearly suggested that the extracted sample was xylan fraction.

### Optimization of xylooligosaccharide (XOS) production

The influence of enzyme (xylanase) concentration, incubation time, and xylan concentration on XOS production was evaluated. The predicted XOS production of all the center points (enzyme concentration = 3.75 mg/g of xylan; incubation time = 9 h; xylan concentration = 3%) were 13.19 mg XOS per mL of xylan, whereas the experimental values were varied. About 12.98 ± 0.01, 12.96 ± 0.65, 13.18 ± 0.47, 13.42 ± 0.47, and 13.44 ± 0.24 mg XOS per mL xylan were recovered in run numbers 1, 3, 6, 8 and 14, respectively (Table 4).

**Table 4:** The variable factors and predicted and observed xylooligosaccharide yield

Std.	Run	Factors			Amount of xylooligosaccharide (mg/mL)	
		X1: enzyme concentration (mg/g xylan)	X2: incubation time (h)	X3: xylan concentration (%w/v)	Observed value	Predicted value
16	1	0 (3.75)	0 (9)	0 (3)	12.98 ± 0.01	13.19
12	2	0 (3.75)	1 (16)	1 (5)	17.32 ± 0.79	17.32
13	3	0 (3.75)	0 (9)	0 (3)	12.96 ± 0.65	13.19
7	4	− 1 (1.25)	0 (9)	1 (5)	16.94 ± 0.32	16.95
9	5	0 (3.75)	− 1 (2)	− 1 (1)	13.97 ± 0.14	13.97
17	6	0 (3.75)	0 (9)	0 (3)	13.18 ± 0.47	13.19
8	7	1 (6.25)	0 (9)	1 (5)	17.35 ± 0.31	17.41
15	8	0 (3.75)	0 (9)	0 (3)	13.42 ± 0.47	13.19
3	9	− 1 (1.25)	1 (16)	0 (3)	13.75 ± 0.14	13.75
5	10	− 1 (1.25)	0 (9)	− 1 (1)	14.25 ± 0.31	14.19
1	11	− 1 (1.25)	− 1 (2)	0 (3)	13.05 ± 0.47	13.11
2	12	1 (6.25)	− 1 (2)	0 (3)	13.61 ± 0.14	13.62
11	13	0 (3.75)	− 1 (2)	1 (5)	17.29 ± 0.22	17.23
14	14	0 (3.75)	0 (9)	0 (3)	13.44 ± 0.24	13.19
6	15	1 (6.25)	0 (9)	− 1 (1)	13.88 ± 0.51	13.87
10	16	0 (3.75)	1 (16)	− 1 (1)	14.20 ± 0.03	14.27
4	17	1 (6.25)	1 (16)	0 (3)	13.44 ± 0.16	13.38

The analysis of variance of a quadratic model for the XOS production was shown in Table 5. The regression model for xylan extraction was significant (*P *< 0.0001) with appreciable R^2^ (99.47%) and adjusted R^2^ (98.80%), and non-significant lack of fit (*P* = 0.9278). The results suggested that the model equation was adequate for the prediction of XOS production from xylan by enzyme treatment. The BBD generated a quadratic equation for XOS (Y) production from alkaline extracted xylan of RH was as follows:

**Table 5:** Analysis of variance for quadratic model of xylooligosaccharide production from xylan

Source	Sum of squares	Mean square	F-value	*P*-value
Model	43.62	4.85	146.86	< 0.0001
Residual	0.23	0.033		
Lack of fit	0.023	7.544E−003	0.14	0.9278
Pure error	0.21	0.052		
Cor total	43.85			

Amount of xylooligosaccharides, R^2^ = 99.47%, R^2^ (adjacent) = 98.80%

4 $${\text{Y}} = 1 6. 2 9 2- 0.0 9 8 {\text{X}}_{ 1} + 0.00 5 {\text{X}}_{ 2} - 2. 80 7 {\text{X}}_{ 3} + 0.0 1 4 {\text{X}}_{ 1}^{ 2} + 0.00 4 {\text{X}}_{ 2}^{ 2} + 0. 5 80{\text{ X}}_{ 3}^{ 2} - 0.0 1 3 {\text{X}}_{ 1} {\text{X}}_{ 2} + 0.0 3 9 {\text{X}}_{ 1} {\text{X}}_{ 3} - 0.00 4 {\text{X}}_{ 2} {\text{X}}_{ 3} \ldots .$$

**Fig. 3:**
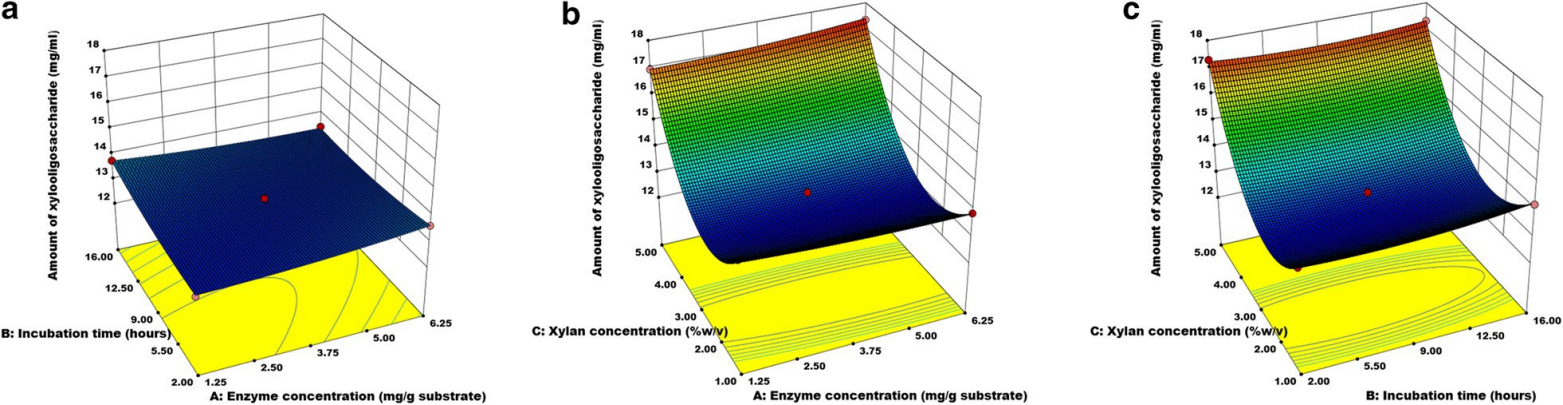
Response surface plot for enzyme hydrolysis of xylan describing the interaction of independent variables such as enzyme concentration, xylan concentration, and incubation time. **a** Effect of enzyme concentration and incubation time, **b** effect of enzyme concentration and xylan concentration, and **c** effect of incubation time and xylan concentration

The estimated regression coefficients revealed that the xylan concentration (*P *< 0.0001) had a strong significant impact on XOS production (Table 6). The interactions of all the variables and its impact on XOS production has been represented as response surface plot (Fig. 3). The results suggested that the enzyme concentration and incubation time exhibited a strong interaction effect (*P *= 0.0468) on XOS production (Fig. 3). The maximum XOS production was observed in the run conditions 7, 2, 13, and 4 with the yield of 17.35 ± 0.31, 17.32 ± 0.79, 17.29 ± 0.22, and 16.94 ± 0.32 mg XOS per mL xylan, respectively.

**Table 6:** Estimated regression coefficients for xylooligosaccharide production from xylan

Terms	Estimated parameters	*P*-value
Enzyme concentration	− 0.098	0.5936
Incubation time	0.005	0.1700
Xylan concentration	− 2.807	< 0.0001
Enzyme concentration × incubation time	− 0.013	0.0468
Enzyme concentration × xylan concentration	0.039	0.0686
Incubation time × xylan concentration	− 0.004	0.5962
Enzyme concentration × enzyme concentration	0.014	0.3457
Incubation time × incubation time	0.004	0.0796
Xylan concentration × xylan concentration	0.580	< 0.0001

## Discussion

XOS has reported for the prebiotic properties, and people are interested in XOS because of its health benefits (Lin et al. 2016). The production of XOS from rice husk (RH) is one of the effective ways to utilize the agricultural wastes. Even though several methods are available to extract xylan from RH, alkaline pretreatment has been used commonly. Alkaline pretreatment accelerates the breakdown of lignocellulosic biomass by smiting the ester bonds in lignin and hemicellulose, which further facilitates the xylan and lignin solubility (Akpinar et al. 2009b; Samanta et al. 2012a).

In this study, NaOH solution was used for xylan extraction, since it showed the higher yield when compared to other strong bases like KOH (Jayapal et al. 2013; Samanta et al. 2012a, b, 2013). The steaming time and steaming temperature are the critical factors that influence the xylan yield. About 54.49 ± 0.61% of xylan yield was observed with alkaline concentration, steaming time, and temperature of 12%, 30 min, and 133.64 °C, respectively (Table 1). To confirm the composition of sugar in alkaline pretreated xylan, the hemicellulose quantification was identified and reported as the XGM content. The highest xylan extraction yield in the run conditions of 12% NaOH concentration, 30 min of steaming time, and 133.64 °C of steaming temperature exhibited the 58.07% of XMG content per total hemicellulose content. The effect of alkaline concentration, temperature, and extraction time on the recovery yield of hemicellulose has been investigated in several previous studies (Nasir and Saleh 2016; Yilmaz et al. 2012; Zhou et al. 2013). In the current study, the increasing NaOH concentration was not significantly increased the xylan extraction yield (*P* = 0.3541). This result is consistent with the increasing NaOH concentration in the range of 10–20% exhibited the xylan extraction yield from 38.4 to 42.5%. However, this increasing of xylan extraction yield did not statistically important (Yılmaz et al. 2012). The previous report suggested that increasing of extraction temperature from 70 to 95 °C were raised exhibited the yield of hemicellulose extraction (Cheng et al. 2011; Zhou et al. 2013). In this study, the increased extraction temperature significantly influenced on extraction yield of xylan in the opposite manner (*P *< 0.0001). Additionally, the increased steaming time significantly reduced the alkaline extracted xylan yield (*P* = 0.0003) which is consistent with the earlier study (Yılmaz et al. 2012). Practically, the confirmation of CCD model usually performed to prove the predicted value in the real situation. The alkaline pretreatment condition was changed as 6.47%, 15 min, and 80 °C of alkaline concentration, steaming time, and steaming temperature, respectively, and the yield of xylan was found as 10.32 ± 0.47%. This actual result exhibited the non-significant data from the predicted value of xylan yield (*P *= 0.4416). The result suggested that the quadratic equation was the effective prediction for the xylan extraction.

The FTIR spectra enable to check the purity and identity of a biomolecule in addition to the indication for the presence of functional groups (Faix 1991; Gonçalves and Ruzene 2001; Ruzene et al. 2008). We performed the FTIR analysis of extracted xylan, and the FTIR pattern showed the high similarity between xylan sample and beechwood xylan (Fig. 2). The rice husk xylan specific bands were observed at 1600–1000 cm^−1^, whereas the specific bands of wheat straw xylan fall in the range of 1100–1000 cm^−1^ region (Ruzene et al. 2008). The broad bands were noticed at a wavelength of 3600–3200 cm^−1^ that was due to the presence of hydroxyl group in the samples. The same band pattern was reported previously (Chaikumpollert et al. 2004; Ruzene et al. 2008). The absorbance at 3422, 2927, 1421, 1251, 1166, 1049, 986 and 897 cm^−1^ are associated with xylan. The bands between 1166 and 1000 cm^−1^ are signature bands of xylan. The bands at 897 cm^−1^ are the indication of C1 group frequency or ring frequency, was observed in extracted xylan and standard xylan. It is the features of beta xylosidic bonds of each sugar monomers (Gupta et al. 1987). The bands between 1200 and 1000, 897 cm^−1^, and around 1046 cm^−1^ were attributed to 4-*O*-methylglucuronoxylan, β-glycosidic linkages between the sugar moieties, and C–O, C–C stretching or C–OH bending in hemicelluloses, respectively. The absence of pectin in the extracted xylan can be substantiated by the absence of band at 1520 cm^−1^ (Kačuráková et al. 1999; Kacurakova et al. 1994; Gupta et al. 1987). The FTIR pattern of the sample revealed that the extracted xylan did not present the pectin in their structure.

The xylan is the desirable substance used for further hydrolysis processes including acid and enzymatic hydrolysis to recover XOS with various degree of polymerization. The enzymatic hydrolysis method has been accepted widely to reduce the use of corrosive chemicals and solvents. Furthermore, acid hydrolysis process needs controlled environment to operate and also produce some unwanted substances like monosaccharide and furfural. Due to the specificity of the enzyme, the unwanted contamination was less in enzyme hydrolysis procedure (Aachary and Prapulla 2011; Samanta et al. 2015).

The maximum XOS production of 17.35 ± 0.31 mg XOS per mL xylan was observed in the run conditions of 6.25 mg per g xylan of the enzyme, 9 h of incubation time, and 5% of xylan (Table 4). Several studies have been reported the important factors influenced the production yield of XOS such as pH, incubation temperature, incubation time, enzyme dose, and substrate concentration. In this study, three factors including enzyme dose, substrate concentration, and reaction time are the interesting factors for XOS production by the commercial enzyme. From the previous reports, enzyme dose was varied from 2 to 200 U from XOS production presented in previous studies (Brienzo et al. 2010; Chapla et al. 2012; Gowdhaman and Ponnusami 2015; Jayapal et al. 2013; Samanta et al. 2014; Siti-Normah et al. 2012). The increasing of enzyme concentration from 2.65 to 13.25 U were significantly increased the reducing sugars after enzyme hydrolysis (Samanta et al. 2014, 2016). In the present study, the decreasing enzyme concentration exhibited the decreased XOS yield. However, the enzyme dose did not a significant factor after the estimated regression coefficients analysis (*P *= 0.5936). Incubation time is one of the factors that directly influenced by XOS production yield. Similar to this study, the increasing incubation time showed the raised of XOS production yield (Samanta et al. 2014, 2016). The effect of substrate concentration for XOS production was carried out by different concentration of xylan. When xylan concentration was increased, the XOS production yield significantly reduced (*P *< 0.0001). This result agreed with the previous observations of other authors revealed that the dissolution property of xylan reduced when increasing the substrate concentration reflected the decreased of XOS yields, owing to decrease the enzyme activity by the present of impurities in the substrate as well as increase viscosity of substrate solution. Furthermore, the reduction of water content in the medium presented in high concentration of xylan reflected the decreased in XOS production yields (Gowdhaman and Ponnusami 2015; Siti-Normah et al. 2012). The confirmation of BBD model was proved by changing the conditions for XOS production as enzyme concentration (1.25 mg/g substrate), incubation time (2 h), and xylan concentration (1%), and the actual XOS yield (13.84 ± 0.29 mg XOS per mL xylan) was not significantly different from its predicted value (14.0011 mg XOS per mL xylan) from the quadratic equation (*P *= 0.4249). These indicated that the generated equation (Eq. 4) can be used to predict the optimal operation conditions to produce XOS from xylan.

The xylan was extracted efficiently by alkaline pretreatment coupled with the steam application. The steaming time, steaming temperature, the interaction between steaming temperature and steaming time, and interaction of steaming temperature, were the significant factors that directly influence on recovery yield of alkaline extracted xylan. The FTIR analysis showed a typical signal pattern for the hemicellulosic factions. The XOS production was significantly (*P *< 0.05) influenced by xylan concentration, the interaction between enzyme concentration and incubation time, and interaction of xylan concentration. Also, the results revealed that the xylan extracted from RH as an effective base couple with the steam application and the enzymatic hydrolysis help to maximize the yield of XOS, which can be further used in functional foods and dietary supplements.

## Abbreviations

BBD: Box–Behnken design
CCD: central composite design
DNS: 3,5-dinitrosalicylic acid
FTIR: Fourier-transform infrared spectroscopy
HPLC: high performance liquid chromatography
KOH: potassium hydroxide
NaOH: sodium hydroxide
NREL: National Renewable Energy Laboratory
RH: rice husk
RSM: response surface methodology
XGM: Xylan + Galactan + Mannan
XOS: xylooligosaccharide
